# Gender Difference in Chronic Cough: Are Women More Likely to Cough?

**DOI:** 10.3389/fphys.2021.654797

**Published:** 2021-05-07

**Authors:** Haodong Bai, Bingxian Sha, Xianghuai Xu, Li Yu

**Affiliations:** Department of Pulmonary and Critical Care Medicine, Tongji Hospital, Tongji University School of Medicine, Shanghai, China

**Keywords:** chronic cough, gender difference, estrogen, progesterone, sex hormone

## Abstract

Chronic cough is a common complaint for patients to seek medical cares all over the world. Worldwide, about two thirds of chronic cough patients are females. However, in some regions of China the prevalence of chronic cough between sexes is roughly the same. Estrogen and progesterone can not only have an effect on transient receptor potential vanilloid 1 channel, eosinophils and mast cells, but also influence laryngeal dysfunction, gastroesophageal reflux disease and obstructive sleep apnea hypopnea syndrome, which may lead to increased cough sensitivity in women. On the other hand, the quality of life was adversely affected more in female patients with chronic cough. Both hormones possibly cause gender difference in chronic cough.

## Introduction

Chronic cough is defined as cough lasting no less than 8 weeks. In non-smoker patients not taking protussive drugs and with a normal chest radiograph, the common causes of chronic cough are asthma syndromes (including cough variant asthma and eosinophilic bronchitis), upper airway cough syndrome/postnasal drip syndrome, gastroesophageal reflux-related chronic cough, and atopic cough (Chinese Medical Association of Respiratory Disease Asthma Study Group, 2016). Chronic cough has been one of the main complaints of patients to seek medical cares, which negatively affects the quality of life and its treatment has been a great challenge in clinical practice (Ma et al., 2009; Young and Smith, 2010; Song et al., 2015). In recent years, a gender difference was found in chronic cough. Most studies showed that it was more common in women, and that it was more common in middle-aged and elderly women with refractory chronic cough or unexplained chronic cough (Morice et al., 2014; Yu et al., 2016; Kang et al., 2019). If the mechanism of gender difference could be clarified, it may provide new ideas for the treatment of chronic cough, especially refractory cough, in the future. This article reviews the status of gender difference in chronic cough and possible reasons for it.

## Status of Gender Difference in Chronic Cough

In Europe and America, there is a significant gender difference in patients with chronic cough, a number of studies have shown that the ratio of female to male is 1.86–2.76:1 (French et al., 2005; Kelsall et al., 2009; Morice et al., 2014; Hartley et al., 2015; Campi et al., 2020). In some Asian countries, such as Japan and South Korea, similar results have also been obtained (Niimi et al., 2013; Song et al., 2014; Kang et al., 2019). But in China, it differs in different regions. Studies in Shanghai (Ma et al., 2009; Yu et al., 2009; Ding et al., 2019) and Chongqing (Cao et al., 2009) showed that the proportion of females can reach 60% or more in chronic cough patients, which is consistent with the data of Europe and America, while patients with chronic cough have a roughly equal sex distribution in Guangzhou (Deng et al., 2016; Lai et al., 2019), Lanzhou (Liu et al., 2016), and Beijing (Liu and Lin, 2009). In a national-wide multi-center study, except for a eastern region (Shanghai), chronic cough patients presented an approximately equal gender distribution in western, southern, northern, and northeastern regions of China (Lai et al., 2013; Lai and Long, 2020). What’s more, a female predominance among patients with refractory or unexplained chronic cough has been reported (Ryan et al., 2012; Muccino et al., 2020; Smith et al., 2020) (more specific details about sex distribution are shown in Table 1).

**TABLE 1:** Sex distribution of patients with chronic cough.

**Author/year**	**Region**	**Number**	**Female:Male**	**Average age ($\bar{x}$± S)**
Morice et al., 2014	multi-center	10032	1.92:1	55 ± 14.97
Muccino et al., 2020	multi-center	1314	2.90:1	58.1 ± 12.1
Campi et al., 2020	Italy	1204	2.37:1	61
Smith et al., 2020	United States, United Kingdom	253	3.21:1	60.2 ± 9.9
French et al., 2005	United States	172	2.07:1	53.6
Hartley et al., 2015	United States	128	2.76:1	55.39 ± 13.54
Kelsall et al., 2009	United Kingdom	100	1.86:1	55.8 ± 11
Ryan et al., 2012	Australia	62	1.82:1	61.8
Ding et al., 2019	China (Shanghai)	1311	1.70:1	47.3 ± 15
Ma et al., 2009	China (Shanghai)	110	2.06:1	47 ± 14
Yu et al., 2009	China (Shanghai)	940	1.62:1	49 ± 16
Lai et al., 2019	China (Guangzhou)	1822	1.06:1	43 ± 13.7
Lai et al., 2013	China	704	1.23:1	40.4 ± 12.8
Liu et al., 2016	China (Lanzhou)	173	0.92:1	42 ± 14
Cao et al., 2009	China (Chongqing)	233	1.40:1	44.5
Liu and Lin, 2009	China (Beijing)	103	0.81:1	38 ± 15
Deng et al., 2016	China (Guangzhou)	96	0.96:1	35.4 ± 9.9
Niimi et al., 2013	Japan	313	1.48:1	51.8 ± 18.9
Kang et al., 2019	South Korea	447	1.92:1	54.4 ± 15.9
Song et al., 2014	South Korea	272	2.24:1	52.8 ± 15.7

## Possible Reasons for Gender Difference in Chronic Cough

The gender difference in chronic cough may be related to the increased cough sensitivity in women and more negative impacts on the quality of life in female patients.

### Increased Cough Sensitivity in Women

Cough sensitivity is defined as the reaction intensity of cough reflex to different stimuli. Patients with chronic cough commonly have a clinical characteristic of increased cough reflex sensitivity. Morice (2010) raised a concept of cough hypersensitivity syndrome, defined as a disease with chronic cough as the only or prominent symptom. A number of studies have shown the cough sensitivity of patients with chronic cough is higher than that of healthy people. Both in patients with chronic cough and healthy people, the cough sensitivity of women is higher than that of men (Dicpinigaitis and Rauf, 1998; Kastelik et al., 2002; Song et al., 2014; Lai et al., 2019). Elderly females tend to have higher cough sensitivity (Song et al., 2014; Lai et al., 2019). In the study of Fujimura, among 40 middle-aged females, capsaicin cough threshold in 24 premenopausal subjects was significantly greater than that in postmenopausal subjects (Fujimura et al., 1996).

Cough receptors are nerve endings existing in the tracheal and bronchial epithelial cells and in the basal layer of the epithelium. There are two types. The myelinated Aδ-fiber is sensitive to mechanical stimulus and acid. The main role of Aδ-driven cough is to prevent the aspiration of foreign bodies or reflux. The other one is unmyelinated C-fiber, which is sensitive to chemical substances such as capsaicin and endogenous mediators (Shi and Qiu, 2007; Kavalcikova-Bogdanova et al., 2016). The ability to sense chemical substances of C-fiber depends on the expression of different ion channels. Transient receptor potential vanilloid 1 (TRPV1) is one of them and known as capsaicin receptor, capsaicin and various inflammatory mediators could activate C-fiber through TRPV1. The expression of TRPV1 is increased in the airway of patients with chronic cough (Mitchell et al., 2005), which may be the basis of increased cough sensitivity in such patients. Similar to TRPV1, the main stimulants of transient receptor potential ankyrin 1 (TRPA1) include acrolein, cinnamaldehyde, cold air (<17°C), motor vehicle exhaust and cigarette smoke. Compared with healthy subjects, patients with chronic refractory cough have increased cough sensitivity to allyl isothiocyanate (TRPA1 receptor agonist) and capsaicin. Whether in patients with chronic cough or in healthy subjects, women have more obvious AITC and capsaicin cough sensitivity than men (Long et al., 2019). Purinergic receptor P2X3 is an ATP-gated ion channel which exists in peripheral sensory nerves including the vagal afferent neurons that innervate the cough reflex. A randomized, double-blind, placebo-controlled study from Abdulqawi et al. (2015) showed that P2X3 receptor antagonist could reduce cough frequency of the refractory chronic cough patients. In their opinion, P2X3 receptors seem to have a key role in mediation of cough neuronal hypersensitivity (Abdulqawi et al., 2015).

#### Effects of Estrogen and Progesterone on Cough Sensitivity

The influence of sex hormones on ion channels has been followed for a long time. Estrogen influences TRPV1 activation/sensitization, leading to increased channel excitability (Patberg, 2011; Kavalcikova-Bogdanova et al., 2016), 17β-estradiol can stimulate the expression and release of prolactin, which increases phosphorylation of TRPV1 (Xu et al., 2008). There are some preclinical studies supporting this point. In the research of Pohóczky et al. (2016), the mRNA expression of TRPV1 and TRPA1 in rat endometrium increases significantly after the treatment of diethylstilbestrol, which is a non-selective estrogen receptor agonist. However, no significant change was observed in TRPV1 and TRPA1 expression after combining diethylstilbestrol with progesterone (Pohóczky et al., 2016). Peng et al. (2008) found that the estrus cycle is closely related to activation of TRPV1 channel in pelvic nerves of rats, activation of C-fibers by capsaicin is significantly greater in pro-estrus phase (high level of estradiol and low level of progesterone) than in the metestrus phase (low level of estradiol and high level of progesterone), suggesting estradiol as a hormonal mediator for TRPV1 (Peng et al., 2008). Kiasalari et al. (2010) study showed more than 75% of HE TRPV1 cells express estrogen receptor α in the ganglion of lumbar and sacral regions, suggesting a possible modulating effect of estrogen.

However, this estrogen hypothesis cannot explain the hypersensitive cough in postmenopausal women, who typically have a lack of estrogen. In addition, it was recognized that the activation of TRPV1 by capsaicin can be inhibited by a nonclassical estrogen-signaling pathway (Xu et al., 2008), so it can be speculated that the decrease of estrogen level in postmenopausal women weakens this inhibitory effect and may lead to increased cough sensitivity in middle-aged and elderly women. A study on pain showed that the effect of estrogen on cultured dorsal root ganglion neurons could decrease the expression of P2X3 receptor in both mRNA and protein levels (Ma et al., 2011), suggesting a protective effect of estrogen.

As for clinical studies, Kavalcikova-Bogdanova et al. (2018) conducted a study in healthy women and women taking oral contraceptive pills to analyze the relationship between sex hormones and cough sensitivity during different phases of menstrual cycle. The study showed that capsaicin cough sensitivity is influenced by the level of female sex hormones. Compared with follicular phase, the capsaicin cough sensitivity is higher in luteal phase. And they revealed the correlation between cough sensitivity and the ratio progesterone/estrogen rather than estrogen alone. The progesterone/estrogen ratio is high in luteal phase due to elevation of progesterone and decrease of estrogen. Menopause is characterized by the lack of estrogen, similarly to luteal phase, which may explain increased cough sensitivity in postmenopausal women.

#### Effects of Estrogen on Gastroesophageal Reflux Disease

Gastroesophageal reflux disease (GERD) is a heterogeneous, multi−symptom disease, which is categorized into reflux esophagitis (RE) and non-erosive reflux disease according to endoscopy. In the study for subjects without reflux symptoms or GERD observed by ambulatory 24-h esophageal pH monitoring, women have significantly fewer reflux events at both esophageal measuring spots, and less total reflux time and percentage of time with pH < 4 in the distal esophagus than men (Vega et al., 2013). Epidemiologic studies have indicated that RE is more common in men, while nonerosive reflux disease is more common in women (Asanuma et al., 2016; Kim et al., 2016). The severity and prevalence of GERD seem to be closely related to the reproductive hormone of women, the prevalence of the GERD rises rapidly during the postmenopausal period (Kim et al., 2016). Some studies have shown the incidence of RE increased with age. In addition, old women showed more severe RE than older men, the incidence of severe RE increased higher in postmenopausal women (Furukawa et al., 1999; Menon et al., 2011). It has been revealed that estrogen contributes to tissue resistance in female animal models via anti-inflammatory activity. Masaka et al. (2013) demonstrated that estrogen attenuates RE by inactivating mast cells, using a rat model of surgically induced RE. Moreover, esophageal tissue damage in males and ovariectomized rats was attenuated by exogenous 17β-estradiol through weakening mast cell-mediated cytotoxicity and reducing the production of cytokines, especially TNF-α which drives inflammation. And estrogen has been shown to enhance cutaneous wound healing via inactivating macrophages, so anti-inflammatory functions of estrogen may contribute to the gender difference in the incidence of RE (Ashcroft et al., 2003; Gilliver et al., 2011). Reduced levels of estrogen can potentially lead to increased inflammation/immune activation (Grishina et al., 2014) and declining tissue resistance, estrogen in young women may be responsible for GERD being more common in men and postmenopausal women. GERD is also an important cause of chronic cough (Wang et al., 2010; Morice, 2013), so it may be one of the reasons why women, especially elderly women, are prone to cough.

#### Progesterone and Laryngeal Dysfunction

Bucca et al. found that an irritable larynx was very common among patients who presented with chronic cough as the main symptom. Laryngeal hyperresponsiveness was found in more than 2/3 of patients with chronic cough due to perennial rhinitis/chronic rhinosinusitis, GERD and unknown causes (Bucca et al., 2011). Randomized trials found that the speech therapy which ameliorates laryngeal dysfunction is also effective in patients with chronic cough, suggesting a potential connection between laryngeal dysfunction and chronic cough (Gibson and Vertigan, 2009; Ryan et al., 2009). The gender difference in patients with chronic cough caused by laryngeal dysfunction is still unclear, and further studies are needed to confirm it. Increased concentration of progesterone could rise laryngeal edema and venous dilatation. It may activate the rapidly adapting receptors in airways, which are highly sensitive to the changes of pulmonary extravascular space. Activation of rapidly adapting receptors ensues respiratory stimulation and cough (Kavalcikova-Bogdanova et al., 2016, 2018).

#### Effects of Estrogen and Progesterone on Inflammation

The increased cough sensitivity of patients with chronic cough is closely related to the increased expression and activation of TRPV-1 in airway epithelial nerves (Chuang et al., 2001). Airway inflammation in patients with chronic cough is caused by infiltration of inflammatory cells such as eosinophils, neutrophils, and lymphocytes (Grabowski et al., 2013), which sensitizes TRPV-1 receptors by releasing relevant inflammatory mediators, including bradykinin and lipoxygenase metabolites of arachidonic acid (Chuang et al., 2001; Ricciardolo, 2001; Morice and Geppetti, 2004). At the same time, airway inflammation increases the level of protons, neurotransmitters, and growth factors in the local environment (Morice and Geppetti, 2004), such as brain-derived neurotrophic factor and bradykinin, which induce thermal response and contribute to the activation of TRPV-1 (Woolf and Salter, 2000). In addition, a related study has shown that the mechanical stress on the airway from severe air flow caused by cough could lead to the mechanical injury to airway (Irwin, 2006), resulting in the shedding of airway mucosal epithelium, goblet cells and squamous cells metaplasia, and infiltration of lymphocytes and plasma cells (Irwin et al., 2006). Besides, changes in the airway pressure caused by cough could lead to enhanced the interaction between white blood cells and endothelium in the trachea and the recruitment of white blood cells by affecting tracheal vascular endothelium (Lim and Wagner, 2003). It was documented that estrogen and progesterone receptors are expressed in mouse, rat and human mast cells (Jensen-Jarolim and Untersmayr, 2008), β-estradiol significantly enhanced the eosinophil adhesion to human mucosal microvascular endothelial cells, and eosinophils stimulated by a combination of β-estradiol and progesterone showed significantly induced degranulation (Hamano et al., 1998b), estradiol and progesterone can also activate mast cells. Hamano et al. (1998a) found that estradiol and progesterone increased the expression of H1 receptors in human nasal epithelial cells and microvascular endothelial cells leading to enhanced nasal hyperreactivity. Bowser and Riederer (2001) believed that progesterone may affect fibroblasts and extracellular matrix in the nasal mucosa of pregnant women, and estrogen and progesterone can change the concentration of neurotransmitter P, leading to nasal congestion and other symptoms. Postnasal drip from nasal diseases is one of the causes of chronic cough (Chinese Medical Association of Respiratory Disease Asthma Study Group, 2016). The effect of estrogen and progesterone on allergic mediators such as eosinophils, mast cells, histamine, and the inflammatory mediators affect may be one of the possible mechanisms of increased cough sensitivity in women. However, we have also noted that late-onset asthma often present with eosinophilic inflammation more frequently in men than women (de Groot et al., 2016). Some asthma cluster analyses have mentioned a higher proportion of men with late-onset asthma mainly characterized by eosinophilic inflammation and fewer symptoms (Haldar et al., 2008), but no corresponding gender difference has been observed in other studies of related asthma cluster analyses on late-onset asthma patients (Moore et al., 2010; Lefaudeux et al., 2017), suggesting that female hormones do not necessarily cause more inflammation than men. Therefore, the above speculation needs to be confirmed by more rigorous basic research.

#### Obstructive Sleep Apnea Hypopnea Syndrome and Cough Sensitivity

More attention has been paid to the relationship between obstructive sleep apnea (OSA) and chronic cough. It was documented that cough sensitivity to capsaicin increased in obstructive sleep apnea hypopnea syndrome patients (Shi et al., 2019). Obstructive sleep apnea hypopnea syndrome is one of the causes of chronic cough. Gastroesophageal reflux, postnasal drip and airway inflammation are possible mechanisms of chronic cough induced by OSA (Liang et al., 2012). Epidemiologic studies showed that, OSA was two to three times more prevalent in men than in women before age 50, while the prevalence of OSA increased in postmenopausal women, who are 3 times more likely to have OSA compared to before. The risk of OSA also increased in women during pregnancy or with polycystic ovarian syndrome (Galvan et al., 2017). Lower levels of estradiol might contribute to the increased prevalence of OSA during the menopause transition and early postmenopause. There are studies demonstrating an inverse relationship between estradiol and OSA, which is indirectly suggested by reports that postmenopausal women using hormone therapy have a lower prevalence of OSA than those not taking hormone therapy (Bixler et al., 2001; Shahar et al., 2003; Galvan et al., 2017). OSA may be one of the reasons for increased cough sensitivity in postmenopausal women.

#### Central Neural Circuits Regulating Cough

Sterusky et al. (2020) have found that female guinea pigs respond the same way to the commonly used tussive agents (capsaicin, distilled water, allyl isothiocyanate, cinnamaldehyde, and citric acid) as male guinea pigs, no obvious differences in cough count and cough latency were observed between female and male guinea pigs. In their opinion, there is not any huge difference in peripheral sensory pathways of cough between humans and guinea pigs, so something different may exist in the central neural circuits regulating cough, which may be responsible for the gender difference of chronic cough in human. Morice et al. (2014) also found that females were more sensitive to capsaicin challenge, when got lower stimulus, the magnitude of the activation in the somatosensory cortex of females was approximately twice that of males in fMRI, the preponderance of females in chronic cough may be explained by sex-related differences in the central processing of cough sensation. At present, the explanation for possible gender differences in cough sensitivity of central sensitization is limited, but we can learn from research about pain. Both chronic cough and chronic pain are mediated by unmyelinated C and myelinated Aδ sensory fibers (Bolser et al., 2006), exhibiting a similar neurobiological properties (O’Neill et al., 2013). They are regulated by cerebral cortical activity (Mazzone et al., 2013). Women are more sensitive to and less tolerant to pain than men (Sorge and Totsch, 2017). In addition, women are more sensitive to central sensitivity than men, showing an enhanced response of nociceptors to normal or subthreshold harm inputs (Smith et al., 2019). However, few studies have been conducted to explore the mechanism of gender differences in central sensitization of chronic cough. Thus, further studies are needed. One other reason why there was no gender bias in the cough of guinea pigs, might be the guinea pigs in the research were just 7 weeks old. The average lifespan of guinea pigs is 4–5 years, and female guinea pigs typically begin to develop follicles at 14 days and ovulate at about 60 days, the 7-week-old guinea pigs have not yet reached middle age and were not significantly affected by sex hormones, so there was no sex difference in cough of guinea pigs.

### Impaired Cough Suppression

Recent studies have shown that impaired cough suppression may be one of the reasons why symptoms in chronic cough patients cannot be controlled. Cho and Hilton et al. (2020) revealed that patients with refractory chronic cough were less able to voluntarily suppress capsaicin or other noxious stimuli-evoked cough compared to healthy controls (Cho et al., 2019). In functional brain imaging study involving patients with refractory chronic cough, Mazzone (2019) found a reduced level of activity in component regions of their cough suppression network in the inability of patients to suppress cough. Whether there is any gender difference in cough suppression is unclear, which needs to be further investigated.

### The Quality of Life in Female Patients With Chronic Cough Decreased Significantly

Chronic cough has negative effects on health-related quality of life (HRQOL) of patients in many aspects including physical, psychological, and social domains (Ma and Qiu, 2008; Ma et al., 2009). For most patients with chronic cough, the main reason for seeking medical attention is often a serious decline in the quality of life (Yang et al., 2010). Won et al. (2020) investigated the relationship between chronic cough and HRQOL using the 3-level EuroQoL 5-dimension component index score. They found that chronic cough was obviously associated with HRQOL among adults more than 40 years old, the overall 3-level EuroQoL 5-dimension component index score was significantly lower in patients with chronic cough, and chronic cough had a greater impact on HRQOL in women more than 65 years old (Won et al., 2020). Our previous study showed that several adverse events including embarrassment, frustration and sleep disorder were more apparent in women than men as measured with Leicester cough questionnaire although there was no confirmed gender difference in overall HRQOL of patients with chronic cough (Ma et al., 2009). Due to the different characteristics of physiological structure, stress urinary incontinence is more prevalent in women than men. It was reported that more than 50% of female patients suffered from stress urinary incontinence due to chronic cough, while the proportion of male patients is far lower (Yang et al., 2010). The discomfort, embarrassment and anxiety caused by urinary incontinence can limit women’s social activities and seriously affect their quality of life. The serious decline in quality of life leads to a higher rate of female patients seeking medical attention, females are more likely to refer to specialist clinics than males due to stronger perception of discomfort, which is also an important reason for the gender difference of chronic cough.

## Possible Explanation for a Roughly Equal Sex Distribution in Some Regions

As mentioned above, there are regional differences in the gender distribution of chronic cough patients in China. The reason has not yet been clearly explained, which may be related to the different patients enrolled in various studies and different healthcare systems.

Age difference: In Table 1, the average age of the patients with chronic cough is over 51 in occident, Japan and South Korea, from 44.5 to 49 in Shanghai and Chongqing, and 35–43 in Guangzhou, Lanzhou and Beijing. That is to say, patients in the studies that have a roughly equal sex distribution are younger. Cough sensitivity is generally higher in females, especially in elderly females (Song et al., 2014). A roughly equal sex distribution in studies of Guangzhou and other places may be due to the younger age of enrolled patients.

Exclusion criteria difference: Smoking is an important risk factor for chronic cough (Çolak et al., 2017). Current smokers or ex-smokers who stopped smoking less than 2 years before the first visit to the clinic were excluded from our studies (Ma et al., 2009; Yu et al., 2009; Ding et al., 2019), while smokers were not excluded or ex-smokers who stopped smoking only less than 6 months were excluded in some studies (Lai et al., 2013, 2019). There are more male smokers in China, which may have an impact on the results of studies and cause regional differences in the gender distribution.

Healthcare system difference: In Europe and America, patients need strict referral from family doctors and general practice before going to specialist clinics. However, patients in China can freely choose hospitals for treatment and go directly to specialist clinics. Therefore, the data used in Chinese studies were close to general population in some degree, while general population studies did not find a consistent female predominance in chronic cough (Chen et al., 2006). It may be one of the reasons for a roughly equal sex distribution in some regions of China.

As for climate and occupation, there is a lack of multicenter investigations studying the influence of climate and occupation on gender distribution of chronic cough patients in China. Further investigations are needed to clarify it.

## Conclusion

**FIGURE 1:**
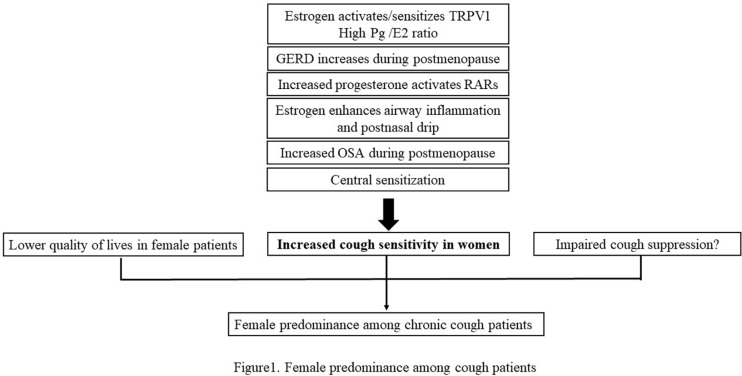
Female predominance among cough patients.

The gender difference is an important characteristic of chronic cough. Most studies show that it is more common in women, and the same as refractory/idiopathic chronic cough. Estrogen and progesterone can not only have an effect on TRPV1 channel, eosinophils and mast cells, but also influence laryngeal dysfunction, GERD, and obstructive sleep apnea hypopnea syndrome, which may lead to the increased cough sensitivity in women. And the quality of lives was adversely affected more in female patients. Increased cough sensitivity in women and more negative impacts on the quality of lives in female patients are possible reasons for the gender difference in chronic cough. The regional differences in the gender distribution of chronic cough patients in China may be related to the different patients enrolled in various studies. In the future, more studies are needed to determine the specific mechanisms leading to gender difference of chronic cough, which may provide new ideas and targets for the treatment of refractory cough (the main content is summarized in Figure 1).

## Author Contributions

XX and LY conceived and critically reviewed the structure of the manuscript. HB drafted and revised the manuscript. HB and BS collected the literatures and completed the tables. All authors read and approved the final manuscript.

## Conflict of Interest

The authors declare that the research was conducted in the absence of any commercial or financial relationships that could be construed as a potential conflict of interest.

## Abbreviations

TRPV1: transient receptor potential vanilloid 1
GERD: gastroesophageal reflux disease
TRPA1: transient receptor potential ankyrin 1
RE: reflux esophagitis
OSA: obstructive sleep apnea
HRQOL: health-related quality of life.
